# Macrophage Polarization States in the Tumor Microenvironment

**DOI:** 10.3390/ijms22136995

**Published:** 2021-06-29

**Authors:** Ava J. Boutilier, Sherine F. Elsawa

**Affiliations:** Department of Molecular, Cellular and Biomedical Sciences, University of New Hampshire, Durham, NH 03824, USA; ab1274@wildcats.unh.edu

**Keywords:** tumor-associated macrophage, tumor microenvironment, metastasis, cytokine signaling, polarization, malignancy

## Abstract

The M1/M2 macrophage paradigm plays a key role in tumor progression. M1 macrophages are historically regarded as anti-tumor, while M2-polarized macrophages, commonly deemed tumor-associated macrophages (TAMs), are contributors to many pro-tumorigenic outcomes in cancer through angiogenic and lymphangiogenic regulation, immune suppression, hypoxia induction, tumor cell proliferation, and metastasis. The tumor microenvironment (TME) can influence macrophage recruitment and polarization, giving way to these pro-tumorigenic outcomes. Investigating TME-induced macrophage polarization is critical for further understanding of TAM-related pro-tumor outcomes and potential development of new therapeutic approaches. This review explores the current understanding of TME-induced macrophage polarization and the role of M2-polarized macrophages in promoting tumor progression.

## 1. Introduction

Macrophages are myeloid cells that are essential members of the innate immune response [1]. These heterogenous cells originate from monocyte precursors in the blood and differentiate in the presence of cytokines and growth factors in the tissues they infiltrate [2,3]. Macrophages are found in every human tissue in the body and exhibit anatomical and functional diversity [4]. These cells have three key functions: phagocytosis, exogenous antigen presentation, and immunomodulation through cytokine and growth factor secretion. As one of the earliest immune cells to encounter antigens at the site of infection or injury, their response is critical to the remainder of the immune response. Antigen-presentation, the ability to phagocytose, digest, and present antigen through the major histocompatibility complex class II (MHC II) system, is crucial for the activation of the adaptive immune system and highlights one of the key roles that macrophages play in the immune response [5]. T-cells (specifically T-helper cells) can recognize these MHC II complexes on classically activated macrophages through their T-cell receptors (TCRs), leading to further activation of the adaptive immune response [5]. Through phagocytosis, macrophages are also able to assist in the resolution of inflammation by effectively eliminating pathogen materials as well as assist in the clearance of apoptotic host cells such as neutrophils [6]. Without macrophages at the site of infection/tissue damage, an elevated apoptotic neutrophil population and elevated and prolonged inflammation has been demonstrated [7]. Macrophages are not only responsible for phagocytosis of foreign antigen; they also coordinate processes that initiate new tissue formation of the extracellular matrix and new blood vessel formation through angiogenesis under normal physiological conditions [4,8]. Macrophages, therefore, play a key role in tissue homeostasis under normal physiological conditions as well as after tissue damage. Macrophages also make key hematopoietic decisions by engulfing cells exuded from the bone marrow that do not express the CD47 ligand [9]. Macrophages engulf dead cells after infection or injury through recognition of the phosphatidylserine that is externalized on apoptotic cells. Phosphatidylserine is typically confined to the inner plasma membrane, but during apoptosis, it becomes externalized on the cell surface [10]. The engulfment of neutrophils and erythrocytes in the spleen and liver resolves the problems of neutropenia, splenomegaly, and reduced body weight [6].

Macrophages also regulate angiogenesis through different mechanisms. Macrophages can identify vascular endothelial cells and instruct them to undergo apoptosis if they do not receive countersignals from pericytes to survive. Macrophages have been shown to instruct functional angiogenesis in normal vessel maturation [11], wound healing [12], and development [13] and nonfunctional angiogenesis in many types of cancer [14] and chronic inflammatory conditions [15]. The protein, Wingless-Type MMTV Integration Site Family Member 7B (WNT7B), is secreted by macrophages and triggers apoptosis of vascular endothelial cells and, in the absence of WNT7B-secreting macrophages, there is significant vascular overgrowth. WNT7B secretion by macrophages is triggered by the presence of ANG2 secreted by mature blood vessels [13]. ANG2 secretion by blood vessels causes macrophages to upregulate WNT7B, which induces the proliferation of vascular endothelial cells and allows them to be targeted by ANG-2-induced apoptosis [13]. In response to *Wnt7b*, vascular endothelial cells enter the cell cycle and die in the G1 phase due to ANG2-mediated withdrawal of survival signals [16]. To induce angiogenesis, macrophages secrete WNT11 and WNT5A, which induce the expression of soluble vascular endothelial growth factor (VEGF) receptor 1 (VEGFR1) in an autocrine fashion. Soluble VEGFR1 regulates the levels of VEGF so vascular complexity is reduced, and the vasculature is more properly organized [17]. Wnt5a and Wnt11 are associated with non-canonical Wnt signaling. In a study of angiogenesis regulation by the non-canonical *Wnt–Flt1* pathway, soluble and membrane-tethered VEGFR1 was measured by quantitative PCR in the RAW264.7 myeloid cell line after *Wnt* treatment, and both soluble and membrane-tethered VEGFR1 expression were significantly increased after RAW264.7 treatment with *Wnt5a* [17]. Macrophages are not limited to just blood vessel formation, but also play a key role in lymphangiogenesis during development and inflammation [18]. Macrophages can promote lymphangiogenesis by transdifferentiating into lymphatic endothelial cells and becoming incorporated into growing lymphatic vessels or by secreting growth factors and proteases that lead to lymphatic vessel formation [18]. Lymphangiogenesis can lead to tumor metastasis [19], making the role of macrophage regulation important to understand and further investigate.

## 2. Macrophage Markers

Regardless of origin, the major lineage regulator of almost all macrophages is the macrophage colony-stimulating factor 1 receptor in both humans (CSF1R) and mice (Csf1r). This is a class III, transmembrane tyrosine kinase receptor that is expressed on most mononuclear phagocytic cells [4]. While CSF1R/Csf1r is important in macrophage differentiation, *Csf1r*^−/−^ mice have still exhibited some tissue macrophages, indicating the importance of other macrophage growth factors such as granulocyte–macrophage colony-stimulating factor (GM-CSF) and IL-3, which act as macrophage growth factors in tissue culture [4]. Macrophages also constitutively express the surface marker F4/80 in mice [4,13] and the equivalent constitutive markers CD14 and CD68 in humans [20,21,22,23].

### 2.1. Human Macrophage Markers

In humans, M1 macrophages are typically identified by the surface markers CD86 and CD64 [23,24]; the macrophage receptor with collagenous structure MARCO [23,25,26]; C-X-C chemokine ligand (CXCL) 9, 10, 11 (CXCL9, 10, 11) [23]; nitric oxide synthase 2 (NOS2), the suppressor of cytokine signaling 1 (SOCS1); and secretion of IL-6, IL-12, IL-1α [27], and TNF-α [24]. M2 macrophages typically express the surface markers CD206 [23,24,25,28,29] and CD163 [23,29] and express/secrete transforming growth factor-beta (TGF-β), peroxisome proliferator-activated receptor gamma (PPARγ), C-C motif chemokine ligands 14 and 22 (CCL14 and CCL22) [28], and arginase-1 (ARG-1) (Table 1) [18,22].

### 2.2. Mouse Macrophage Markers

While most macrophage markers are the same in both humans and mice, some exceptions exist. Murine M1 macrophages express macrophage receptors with collagenous structures (Marco) [23,25,26], Cxcl9, Cxcl10, Cxcl11 [23], Nos2, and Socs1, and secrete Il-6, Il-12, Il-1α [27], and Tnf-α [24], all comparable to their human counterparts. Murine M1 macrophages do not express surface markers CD86 or CD64. Murine M2 macrophages typically express the surface markers Cd206 [17,18,19,21,22] and Cd163 [23,29] and express/secrete Tgf-β, Pparγ, Ccl14 and Ccl22 [28], and Arg-1 [24,29], similar to human M2 macrophages; however, they also have Chitinase-3-like protein 3 (Chil3), unique from human M2 macrophages (Table 1) [29].

## 3. Macrophage Polarization

**Figure 1:**
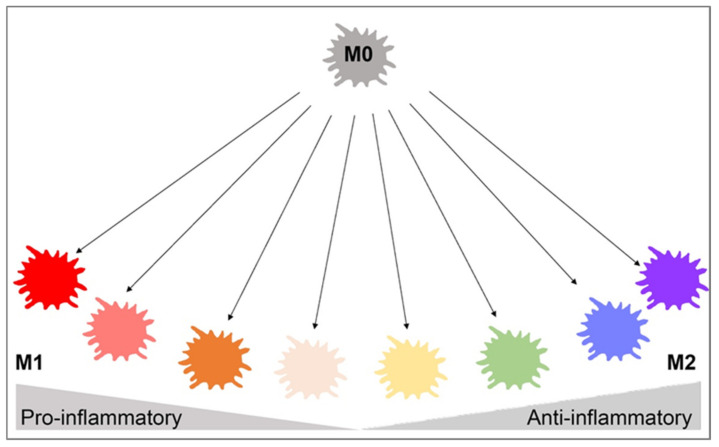
Macrophage polarization gradient. This figure illustrates the heterogeneity of macrophage polarization in place of binary M1/M2 classifications. The pro-inflammatory M1 and anti-inflammatory M2 cells lie on opposite ends of the polarization axis, but many macrophages with mixed pro- and anti-inflammatory characteristics exist in between. Environmental changes may cause macrophages to shift from M1 to M2, vice versa, or to a hybrid of both cells. This highlights the plasticity of macrophages and interdependence on the surrounding environment. This figure was created with Biorender.com (accessed on 1 May 2021).

Many phenotypes of macrophages have been characterized based on their in vitro characteristics in cell culture experiments. Primarily, the classically activated M1 phenotype and the alternatively activated M2 phenotype are differentiated based on different surface receptor expression, secretory profiles, and functions [4,30]. Recent studies of gene expression of in vivo wound healing have shown that macrophages exhibit a pro-inflammatory M1 secretory profile during the early stages and then transition to an anti-inflammatory M2 gene expression profile during the later healing stages [31]. Macrophage polarization refers to the activation state of a macrophage at a singular point in time, but due to the plasticity of macrophages, their polarization state is not fixed and can be altered based on the integration of multiple signals from other cells, tissues, and pathogens [32]. While macrophage polarization is typically discussed as a singular point in time, it is important to understand that M2-type macrophages can switch to an M1 phenotype, or vice versa, based on environmental changes such as cytokine and growth factor secretion, inflammation, infection, injury, hypoxia, and other conditions. Macrophage polarization is more complex than the M1 and M2 binary classification, with those subtypes representing the extremes on the spectrum of macrophage polarization (Figure 1). Many of these subsets express combinations of M1 and M2 cell markers and have yet to be formally defined.

### 3.1. M1 and M2 Macrophages

M1 macrophages are pro-inflammatory in nature and are characterized by their high capacity to present antigens, produce interleukin 12 and 23 (IL-12 and IL-23) [33], and activate type-I T-cell responses [5]. They inhibit cell proliferation and cause tissue damage through the secretion of pro-inflammatory cytokines and nitric oxide (NO) and are induced by T-helper type-1 cytokines including interferon-γ (IFN-γ), interleukin-1β (IL-1β), and lipopolysaccharide (LPS) [1,3,4,34]. M2 macrophages are typically anti-inflammatory in nature and are characterized by their poor ability to present antigen; having low IL-12 and high IL-10, IL-4, and IL-13 secretory profiles; and immunosuppressive effects [5]. These cells promote cell proliferation, tissue repair, angiogenesis, and phagocytosis to downregulate inflammation and “clean up” after inflammatory events and are T-helper type-2 activators and TH1 inhibitors [1,3,4,5,34]. While macrophage polarization is often defined as a specific moment in time, it is important to note that these markers are often present on many subtypes of macrophages in varying expression levels. For example, M2 macrophages can still express M1 markers but with lower levels than M1 macrophages, and vice versa [35].

### 3.2. Extrinsic Polarization

Extrinsic polarization is a primary method of macrophage polarization and is mediated by cytokine secretion by other cells such as CD4^+^ T_H_1 or T_H_2 cells (Table 2). Some non-cytokine, extrinsic pathways of macrophage polarization do exist, however, including hypoxia as well as the production of lactate within tumors, which drive M2 polarization [36].

**Figure 2:**
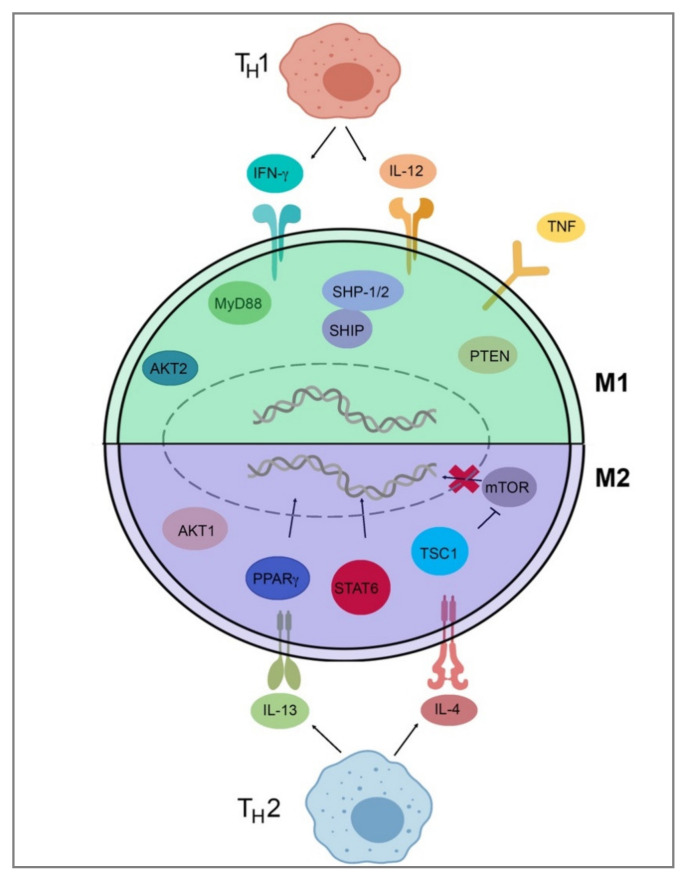
Signal pathways of macrophage polarization. This figure illustrates several of the various mechanisms that drive extrinsic macrophage polarization. Those pathways include IFN-y and IL-12 secretion by TH1 T-cells, LPS signaling through mTOR/Akt or TLR4, Akt2/NF-κB activation, SHP-1/2 inhibition of Cd11b, SHIP and MyD88 inhibition of M2 genes, PTEN activation, and TNF/TNFR/NF-κB activation to induce M1 gene expression. Induction of M2 genes is directed by the secretion of IL-4 and IL-13 from TH2 T-cells and IL-4Rα receptor activation as well as downstream Stat6-dependent arginase-1 inhibition, PPARγ activation, and TSC1 inhibition of mTOR. This figure was created with Biorender.com (accessed on 1 May 2021).

To detail Table 2 and Figure 2, T_H_1 cells secrete IFN-γ, which drives polarization of macrophages towards an M1 phenotype, while T_H_2 cells secrete IL-4 and IL-13, which drive M2-phenotype polarization [34,37]. IL-4 and IL-13 inhibit the production of nitric oxide, an inflammatory mediator, through the depletion of arginine, which serves as the substrate for iNOS/Nos2. This inhibition of NO production in macrophages was found to be dependent on IL-4 or IL-13 through the depletion of Arg-1 through a Stat6-dependent pathway [38]. This inhibition of NO production leads to a loss of the M1 phenotype and polarization toward the M2 phenotype through cytokines IL-4 and IL-13. IL-4 and LPS signaling can also target the mechanistic target of rapamycin (mTOR) and Akt to trigger polarization. In LPS-mediated M1 polarization, Toll-like receptor 4 (TLR4) activates phosphoinositide 3-kinase (PI3K) followed by Akt and mammalian target of rapamycin complex 1 (mTORC1) activation, leading to M1 polarization [39,40]. Additionally, pharmacological and genetic inhibition of Akt1/2 has shown that Akt1 inhibits M1 activation, and Akt2 leads to the activation of M1 genes, favoring polarization to the M1 phenotype [39,40]. Akt signaling is likely to control macrophage polarization through downstream effectors; for example, Akt signaling inhibits transcription factor Foxo1, which is a key gene in M1 macrophages. Additionally, Akt1 has been implicated as a negative regulator of the nuclear factor, kappa-light-chain-enhanced activity of activated B cells (NF-κB), while Akt2 is a positive regulator. NF-κB is a master regulator of M1 activation [41]. Src homology region 2 domain-containing phosphatase 1/2 (SHP-1/2) inhibits CD11b activity, therefore inhibiting M2 polarization and leading to an increase in M1-type macrophages [42]. Src homology 2 (SH2) domain-containing inositol polyphosphate 5-phosphatase (SHIP) is another phosphatase that inhibits the activation of M2-like macrophages. SHIP^−/−^ peritoneal and alveolar macrophages have been found to be profoundly M2-skewed, with high arginase I levels and impaired LPS-stimulated NO production [43]. Phosphatase and tensin homolog (PTEN) plays a key role in regulating the inflammatory response through M1-polarization. Mice with a myeloid-specific PTEN knockout have been shown to have levels of M2 macrophages and produce lower TNF-α and higher IL-10 in response to TLR ligands, indicating that PTEN plays a key role in M1 macrophage differentiation [44]. Tumor necrosis factor (TNF) has been found to be a positive regulator of M1 polarization through its activation of the NF-κB pathway. Tumor necrosis factor receptor (TNFR) signaling was found to be a negative regulator of M2 polarization in tumor-associated macrophages (TAMs), and myeloid differentiation primary response 88 (MyD88) was shown to suppress M2 gene expression in TAMs, leading to an M1 phenotype [45].

### 3.3. Hypoxia-Induced Polarization

Hypoxia can be a key driver of macrophage recruitment and polarization in the TME. Hypoxia is common in most solid tumors, and TAMs are found in higher concentrations in hypoxic areas. Due to the high concentrations of chemokines, HIF-1/2, and endothelin-2 secreted from hypoxic tissues, macrophages are drawn to the hypoxic areas [46]. Damage-associated molecular pattern (DAMP), high-mobility group box 1 protein (HMGB1) is most commonly associated with hypoxia-induced macrophage polarization. HMGB1 has been shown to be overexpressed in many solid tumors and correlated with the development of hepatocellular carcinoma [47] as well as colon [48] and skin cancers [49]. In metastatic melanoma, serum HMGB1 levels in human patient samples have been shown elevated compared to healthy controls. [50]. Additionally, a murine model of metastatic melanoma analysis of dissociated tumors by flow cytometry showed a significant increase in the total number of TAMs exhibiting a M2 phenotype in HMGB1-positive tumors [50]. Using short-hairpin RNA (shRNA) to target HMGB1, a higher number of M1-polarized macrophages were found at the tumor site, indicating that HMGB1 led to the M2 polarization of recruited macrophages. In the same study, HMGB1 was found to induce IL-10 production in M2-like macrophages through receptor for advanced glycation end product (RAGE)-dependent signaling [50]. HMGB1 had no effect on IL-6, TNF, or IL-1β expression but significantly increased IL-10 expression in bone-marrow derived M2-like macrophages [50]. RAGE^−/−^ mice did not show an upregulation of IL-10 signaling, indicating that this induction was through RAGE-dependent signaling [50]. These hypoxia-associated macrophages secreted higher levels of pro-angiogenic factors VEGF and TNF-α [50].

### 3.4. Intrinsic Polarization

Intrinsic macrophage polarization refers to the origin of the macrophage. Macrophages have classically been described as being derived from bone marrow-derived circulating monocytes. However, additional sources of macrophage progenitors have been discovered. Many organs harbor embryonic-derived populations of resident macrophages that can self-renew and maintain throughout adulthood [4,36]. Most TAMs have been shown to be from either an embryonic precursor (either the fetal liver or yolk sac) or a monocyte precursor from an adult origin. Historically, TAMs have been observed as being exclusively from a circulating monocyte origin that undergoes differentiation upon tissue infiltration, although a higher fraction of resident macrophages have been discovered in solid tumors [51]. TAM recruitment is highly linked to the CCL2/CCR2 axis [52], and in many cancer models, blocking this axis has led to a significant decrease in TAM populations [53]. The theory of monocyte-derived TAMs was tested in a mouse model of pancreatic ductal adenocarcinoma. *Ccr2*^−/−^ mice showed no difference in tumor weight, but a depletion of resident TAMs using an anti-CSF1R antibody and clodronate showed a significant reduction in weight [54], indicating that resident macrophages made up a larger part of TAM populations than previously hypothesized. In mice, embryonic macrophages begin to develop at embryonic day 8 and give rise to macrophages that do not have a monocyte progenitor [4]. The fetal liver serves as the site of hematopoiesis of circulating monocytes originally, but then primary hematopoiesis is shifted to the bone marrow later in development, significantly increasing the bone marrow-derived monocyte population and minimizing the importance of embryonic macrophages [4]. The developmental origin of macrophages has been linked to some changes in polarization state. At any tissue site, there is always a mixture of both bone marrow-derived and embryonic macrophages [55]. The importance of origin in polarization is heavily debated, as hematopoietic depletion in lethal irradiation, chemotherapy, and systemic infection has shown that macrophage populations can fully return from a bone marrow-derived origin [56]. However, data has suggested that bone marrow-derived macrophages are more susceptible to local signals and subsequent polarization than embryonic macrophages, which appear to exhibit less plasticity than BM-derived macrophages [57]. Additionally, tissue signals appear to trump the embryonic developmental signals, and polarization states reflect the signals received from the environment rather than signals received from embryonic macrophages [58].

The mechanism of macrophage polarization is important to understand because of the potential ability to therapeutically manipulate the interchangeable polarization states of macrophages and subsequently promote or inhibit inflammation in cancer and other inflammation-related diseases.

## 4. Inflammation

### 4.1. Role of Macrophages in Inflammation

Macrophages play a key role in the initiation, maintenance, and resolution of inflammation [3]. Inflammation is an integral part of both normal and abnormal physiological and pathological processes and is a body’s essential defense mechanism against damaged tissue or invading pathogens. Inflammation can be triggered by conditions such as infection or injury or can be systemic as seen in type 2 diabetes and cardiovascular disease [59]. Additionally, non-resolving inflammation can be a major driver of disease. Initiation of inflammation is characterized by a rapid influx of neutrophils followed by monocytes that can differentiate into macrophages [8]. Neutrophils, specifically tumor-associated neutrophils (TANs), may influence the polarization of macrophages by releasing chemokines and cytokines, functioning to both recruit and polarize monocytes/macrophages in the TME [60]. Tissue-resident macrophages release IL-8 upon infection, leading to the recruitment of neutrophils. Due to the initially highly inflammatory nature of neutrophils, recent evidence has shown that tissue-resident macrophages may adjust to an M2 phenotype to prevent excess inflammation from occurring [61]. Once the foreign body or injury has subsided, inflammation will begin to resolve. Inflammation can lead to further inflammation through tissue damage and necrosis, making the resolution of inflammation even more critical and highlighting the importance of macrophages in this process [62]. Because of this potential for harm, the inflammatory process is typically tightly regulated and involves external signals such as activation signals IFN-γ, CSF-1, and TNF-α; lipopolysaccharide (LPS); and extracellular matrix proteins that initiate the anti-inflammatory shutdown signals IL-10 and TGF-β that deactivate macrophages and shut down the inflammatory process [3]. An imbalance between these initiation and resolution signals can lead to over- or under-activated inflammation [3]. Even though inflammation can result in tissue damage, especially during chronic inflammation, it is a protective and necessary aspect of the innate immune response, and most cases lead to a normal resolution [8].

Macrophages are key mediators of the entire process of inflammation, and the activation and deactivation of macrophages is normally synchronous with the inflammatory response. When tissues are damaged, inflammatory monocytes are recruited from circulation, migrate into tissues, and differentiate into macrophages. During the initiation and maintenance of inflammation, cytokines such as IFN-γ, GM-CSF, TNF-α, IL-1; lipopolysaccharide (LPS); and extracellular matrix proteins serve as activators of the M1 macrophage phenotype [4]. These M1-type macrophages secrete pro-inflammatory molecules such as TNF-α, IL-6, and IL-12 [4]. If this inflammatory macrophage response is not quickly controlled and mediated, it can become pathogenic and lead to disease progression [31,63]. The resolution of inflammation is led by the deactivation of these inflammatory mediators, permitting either the apoptosis of pro-inflammatory M1 macrophages or the transition to an anti-inflammatory M2 macrophage [64]. The inhibition of inflammation is led by the deactivation of inflammatory mediators, which permits the deactivation of macrophages. This process is spearheaded by M2-type macrophages, which dampen the immune response by secreting anti-inflammatory factors IL-10, TGF-β, and interleukin-1 receptor antagonist (IL-1RA); scavenging debris; and promoting angiogenesis, tissue remodeling, and repair [34,65]. M2 macrophages have a distinct secretory profile responsible for the wound-healing and inflammation-resolving processes, including major roles in extracellular matrix remodeling, angiogenesis, and immune suppression [30].

The significance of macrophage heterogeneity has been under review since the early 2000s and has been linked to many malignancies and autoimmune disorders in recent years [66]. Due to the high plasticity and diversity of macrophages and their role in tissue development, tissue homeostasis, and regeneration, properly functioning macrophages are critical for proper healing after an injury or infection. In some genetic diseases such as systemic metabolic dysregulation and muscle dystrophy, the dysfunction of macrophages can lead to worsening conditions such as cancer, atherosclerosis, fibrosis, and other chronic illnesses [8].

### 4.2. Role of Inflammation in the Tumor Microenvironment

Cancer-associated inflammation contributes to disease development and progression. Inflammation in the TME is linked to leukocyte infiltration and the expression of pro-inflammatory cytokines and chemokines such as IL-1, IL-6, CCL2, CCL5, C-X-C motif chemokine ligand 8 (CXCL8), CD40L, and TNF [5,67,68,69,70]. As previously discussed, inflammation leads to the rapid proliferation of cells. This inflammation, combined with growth factors and DNA-damage promoting compounds such as iNOS, can increase the risk of cancer development and progression [71]. Proliferating cells that sustain DNA damage can continue to proliferate, especially in environments rich in inflammatory cells and growth factors that promote their survival. The cytokine and chemokine balance of pro- and anti-inflammatory mediators is a deciding factor in the progression of a tumor. If there is an abundance of pro-inflammatory cytokines and chemokines, inflammation will increase, and neovascularization and rapid tumor growth will proceed due to the continuation of proliferation, angiogenesis, and survival of cells that have been damaged or altered [71]. Chronic inflammation can lead to the increased production of reactive oxygen species, leading to DNA damage or the loss of cell death or repair programs [71]. While most TAMs produce anti-inflammatory IL-10, this does not impact the pro-inflammatory nature of the TME, as it shunts the ability of cytotoxic T-cells to respond to malignant cells [72].

### 4.3. Influence of the Tumor Microenvironment on Macrophage Polarization

The polarization process of TAMs is directly controlled by cancer cells within the TME [73], and the phenotypic ratio changes drastically as cancer progresses. At early stages, the ratio is more favorable for M1 macrophages, but as cancer cells hijack this process, the M2-like population drastically increases. M1-like macrophages are essential tumor-suppressing cells that initially act in the tumor microenvironment to suppress tumor cell growth [73,74]. M1-like macrophages achieve this suppressing effect by recruiting CD8^+^ T and NK cells to the TME through antigen presentation to the T-cell receptor (TCR) [75] and the tumor-derived chemokine secretion of CXCL9, CXCL10, and CXCL11 to recruit and activate NK cells [76]. These CD8^+^ T and NK cells express high levels of cytokines such as IFN-γ, GM-CSF, and TNF-α as well as chemokines such as CCL4, CCL5, and CCL23 that assist in the further recruitment of immune cells and the signaling of anti-tumorigenic pathways [73,74]. The M1 phenotype is also associated with the expression of IL-12, IL-1, and inducible nitric oxide synthase (iNOS) [77,78]. The M1 phenotype is well-characterized for its anti-tumorigenic properties, and an increased M1/M2 TAM ratio has been linked to an improved 5-year prognosis in ovarian cancers [77].

Most commonly, M1 macrophages are positively associated with longer survival times and most positive clinical outcomes in many cancers such as small cell lung cancer [79], non-small cell lung cancer [80], colorectal cancer [81], ovarian cancer [77], breast cancer [82], oral squamous cell carcinoma [83], and more. However, in some cancers such as renal cell carcinoma (RCC), several markers of M1 macrophages have been found alongside M2 markers in TAMs isolated from patients, indicating that some TAMs can exhibit a hybrid phenotype in some cancers [84]. In the skin, during early stages of tumor development, M1 TAMs shifted to the M2 phenotype in melanoma, but the presence of either M1 or M2 TAMs was associated with poor prognosis [85].

Malignant cells can secrete M2-like cytokines such as IL-10, CCL2/3/4/5/7/8, CXCL12, VEGF, and platelet derived growth factor (PDGF) in order to recruit more monocytes and M0 macrophages to the area and differentiate them into the M2 phenotype [77]. The majority of intra-tumoral macrophages exhibit an M2 phenotype and are correlated with poor prognosis in a number of malignancies [86,87].

Tumor-associated macrophages are implicated in cancer cell latency, growth, and metastasis through the secretion of cytokines, chemokines, and growth factors [66]. In the tumor microenvironment (TME), TAMs are most frequently found in the M2-like, pro-tumor phenotype [66,88,89]. M2-like macrophages characteristically assist the cancer cell in metastasis, angiogenesis, and proliferation through various anti-inflammatory mechanisms.

## 5. Pro-Tumorigenic Outcomes

### 5.1. Immune Suppression

Current literature supports the theory that most TAMs originate from either tissue-resident embryonic macrophages or macrophages derived from circulating monocytes that originate in the bone marrow [87]. Monocytes are recruited to the tumor by various growth factors and cytokines such as CCL2, CCL5, and CSF1 [90,91]. While the tumor microenvironment can polarize both tissue-resident and bone marrow-derived macrophages depending on the tissue type, it is hypothesized that tissue-resident macrophages are the first to be affected [90]. These tissue-resident macrophages primarily cause DNA damage, survival of transformed cells, and cancer-related inflammation. Monocyte-derived macrophages that are recruited to the tumor site usually promote the proliferation and survival of tumor cells and angiogenesis [92].

M2-type macrophages play a significant immunosuppressive role and have been found to secrete immunosuppressive molecules into the TME including IL-10, TGF-β, and human leukocyte antigen G (HLA-G) [93]. Additionally, M2-type cells interact directly with myeloid-derived suppressor cells (MDSC) and actively suppress T-cell-mediated anti-tumor responses [94]. Myeloid-derived suppressor cells are a heterogeneous population of non-defined myeloid cells that typically expand during inflammation, infection, and cancer. In mice, these cells are characterized by GR1 and CD11b expression and in humans, characterized by the phenotype CD14^−^CD11b^+^ [95]. MDSCs are associated with the metabolism of l-arginine, providing the substrate for iNOS and arginase-1. MDSCs express high levels of both iNOS and arginase-1, which both play a direct role in suppression of T-cell function [95]. MDSC are elevated in most individuals with cancer and are key producers of IL-10, reducing the macrophage production of IL-12, skewing macrophages towards the M2-phenotype, and contributing to MDSC suppression [94]. Other cell–cell interactions induce STAT3 activation, which adds many different immunosuppressive cytokines to the TME [96]. M2 macrophages also play a significant role in recruiting regulatory T-cells into the TME through the chemokine receptor CCR4 as well as M2-derived CCL17/CCL22 [96]. M2 TAMs also show increased programmed cell death 1 ligand 1 (PD-L1), also known as B7-H1, and increased cytotoxic T-lymphocyte antigen 4 (CTLA4) ligand expression. Both PD-L1 and CTLA4 are well-characterized immune checkpoints for cytotoxic T-cells, inhibiting their ability to eliminate cancer cells [97,98,99]. In studies from patients with hepatocellular carcinoma (HCC), elevated levels of PD-L1 expression by TAMs correlated with poorer clinical outcomes compared to patients with lower PD-L1 expression [100]. Glioblastoma patient samples showed TAMs having increased expression of PD-L1 compared to circulating monocytes, which had minimal PD-L1 expression. Circulating monocytes with low levels of PD-L1 expression were cocultured with the glioblastoma cancer cell line U251, both through direct contact between cells and by using a 0.2 μm filter. After 24 h, the number of PD-L1 expressing cells increased by more than 2-fold (48.0 ± 5.2% vs. 13.0 ± 3.9%) through the 0.2 μm filter and more than 4-fold (83.9 ± 6.2% vs. 13.0 ± 3.9%) in direct cell-to-cell contact [100]. TAMs were found to dampen PD-L1 expression in tumors and inhibit T-cell infiltration. In a mouse model of Lewis lung carcinoma (LLC), tumor-infiltrating T-cells in LLC grown in mice were analyzed to determine how TAMs affected the defense response. It was found that CD4 and CD8α T-cell infiltration was significantly increased after tumors were depleted of TAMs [97].

Additional immunosuppressive surface ligands expressed by M2 TAMs include the PD-L1 [98] and B7-H4 [99] immunosuppressive surface ligands. In a study of human gastric cancer patient tissue, PD-L1 and B7-H4 expression on circulating monocytes was significantly higher than normal tissue controls, and advanced stage tissues experienced higher levels of B7-H4 expression than earlier stage cancers on circulating monocytes [98]. Further studies showed that B7-H1 expression was significantly higher on TAMs in the gastric cancer tissues than circulating monocytes [98]. B7-H4^+^ TAMs were shown to suppress CD4^+^ T-cell proliferation and IFN-γ secretion more than B7-H4^−^ TAMs [98], leading to greater immune evasion and suppression by TAMs. After surgical removal of the whole gastric tumor, B7-H4 expression decreased substantially, from 7.9 ± 6.9% to 2.8 ± 1.3% one month after surgery [98]. Finally, in vitro, it was demonstrated that gastric cell lines could induce B7-H4 expression on monocytes. Two gastric cancer cell lines, MKN-45 and MKN-74, were directly cocultured with PBMCs for 24 h, resulting in a significant upregulation of B7-H4 expression. In an indirect coculture using supernatants from both cell lines, this upregulation was not observed, indicating that direct cell-to-cell contact was necessary for this induction [98]. TAM-derived prostaglandin E2 (PGE2) initiates the production of CXCL12, which induces myeloid-derived suppressor cell (MDSC) accumulation and inhibits the production of CXCL10, a chemokine that activates anti-tumor immunity [100].

### 5.2. Proliferation

Uncontrollable proliferation is one of the hallmarks of cancer. TAM M2 macrophages express molecules that can directly affect cancer cell proliferation including members of the fibroblast growth factor (FGF) family, namely transforming growth factor beta (TGF-β) and epidermal growth factor (EGF) [89,101]. The epidermal growth factor is a tyrosine kinase that typically plays an important role in normal physiological conditions, causing downstream activation of molecules that allow for the avoidance of apoptosis, the promotion of proliferation and invasion, and metastasis [102]. In many human cancers, EGFR is overexpressed, leading to an increase in cell proliferation, angiogenesis, metastasis, and inhibition of apoptosis [103]. Fibroblast growth factors and their receptors lead developmental signaling pathways responsible for cell survival, migration, and proliferation [103]. TGF-β is a growth regulatory protein that typically inhibits cell proliferation. In cancer, there is a marked upregulation of TGF-β, and this is linked to advanced stages of cancer and decreased survival rates [104]. Cancer cells often lose their response to the inhibitory proliferative effects of TGF-β. The additional functions of TGF-β include angiogenesis and immunosuppressive effects, allowing for immune evasion and metastasis [104].

A study done on the effects of M2 macrophages in an orthotopic nude mouse model of liver cancer showed that M2 macrophages injected into the liver promoted tumor growth, increasing the tumor volume by 3.26-fold compared to the negative control. Injected M1 macrophages showed a 2.31-fold decrease compared to the control [30].

### 5.3. Lymphangiogenesis, Angiogenesis, and Metastasis

Angiogenesis is an essential process for the survival of malignant tissue, providing nutrients and oxygen for growth. While angiogenesis theoretically provides all necessities for tumor survival, tumor angiogenesis is not a perfect process, leading to many dysfunctional vessels and the perpetuation of hypoxia [105]. TAMs play a key role in tumor angiogenesis and have been described in animal models of ovarian cancer, cervical cancer, prostate cancer, breast cancer, and melanoma [101]. TAMs can sense hypoxia in tumors and react with the production of VEGFA, which can stimulate the chemotaxis of endothelial cells and macrophages and lead to an elevated expression of MMP9 from TAMs [101]. This elevated MMP9 mediates extracellular matrix degradation and the release of bioactive VEGFA [101]. Hypoxia is strongly associated with adverse prognosis in cancer, and hypoxia pathways are frequently activated during cancer development. In non-small cell lung cancer (NSCLC), TAMs were shown to enhance tumor hypoxia in mouse subcutaneous tumors and in patients. In mouse models of NSCLC, TAMs exhibited increased gene expression in hypoxic pathway-signaling molecules Vegfa, Slc2a1, Pdk1, and Cxcr4. Interestingly, M1 marker Nos2 was upregulated, and M2 marker arginase-1 was also upregulated, indicating a mixed phenotype in angiogenesis-promoting TAMs [104]. TAMs can also influence hypoxia in the TME because of their aberrant, pro-angiogenic factor secretion, leading to leaky blood vessels that lose normal structure and function. These blood vessels are often leaky, with loose endothelial junctions, defective basement membranes, and lacking pericyte coverage. Macrophages and hypoxia exist in a positive feedback loop, as hypoxia drive TAM polarization, and TAMs drive hypoxia through poor vessel formation [100].

The epithelial–mesenchymal transition (EMT) is a process where epithelial tumor cells lose their epithelial characteristics and gain mesenchymal function [106]. This transition contributes to overall metastasis through increased invasiveness and motility of the cancer cells themselves [107]. This EMT process enables cancer cells to leave the tissue site, enter the bloodstream, and infiltrate other body sites.

M2-like macrophages play an important role in EMT during cancer progression [108]. They are able to induce EMT through various signaling pathways such as the TLR4 and IL-10 pathways [109], the TGF-β/Smad2 pathway [109], and the miR-30a/NF-κB/Snail [110] signaling pathways. Additionally, high expression of M2 marker CD68 has been linked to loss of E-cadherin expression, an essential tumor suppressor protein that prohibits EMT and metastasis [106].

M2 macrophages play a key role in the initiation of metastasis through the secretion of pro-angiogenic cytokines and growth factors [111] (Table 3). Neovascularization of the tumor microenvironment is crucial for not only nutrient supply, but also for initiation of metastasis, as cancer cells can enter the bloodstream and travel throughout the body to establish foothold in other areas. The angiogenic involvement of M2 macrophages can be further subcategorized into M2a, or alternatively activated macrophages, and M2c, or regulatory macrophages. M2a-induced angiogenesis is regulated by fibroblast growth factor (FGF) signaling, and the M2c-induced angiogenesis is regulated by placental growth factor (PlGF) signaling [111]. TGF-β also plays a key role in the angiogenic progression of malignant cells. Early in tumor development, TGF-β is a tumor suppressor factor and it inhibits proliferation and induces apoptosis. Tumor cells eventually overcome the TGF-β-induced suppressive effects, and TGF-β induces the epithelial–mesenchymal transition, (EMT) which facilitates invasion and metastasis. Overexpression of TGF-β is reported in many human cancers and is constitutively expressed by M2 TAMs. This overexpression is correlated with tumor progression, metastasis, angiogenesis, and poor prognosis [112]. Additionally, M2-like macrophages promote blood vessel formation through their close association with endothelial cells in the TME. M2-macrophages have been found to co-localize with these endothelial cells at the branching points and merge into tubes to become part of the tubular network [111].

TAMs support tumor lymphangiogenesis by secreting pro-lymphangiogenic factors and by trans-differentiating into lymphatic epithelial cells. TAMs produce matrix metallopeptidase 9 (MMP9) abundantly, which leads to the development of lymphatic vessels [101] in addition to VEGFR-3 and its ligands VEGF-C and VEGF-D, leading to lymphangiogenesis. TAMs can also integrate directly into peritumoral lymphatic vessels, where they lose their macrophage functions and become a part of the lymphatic vessel wall. Indirectly, TAMs produce the enzymes plasmin, urokinase plasminogen activator, and MMP, which regulate matrix remodeling and growth factor regulation [5,113]. Similar to angiogenesis, lymphangiogenesis provides an additional avenue for malignant cells to travel through the body and establish footholds in other areas [114].

Both lymphangiogenesis and angiogenesis can play key roles in the initiation of metastasis, allowing malignant cells to travel via new formed vessels to various tissues in the body and causing complications in therapy as well as decreased survival [103,114]. Understanding the role of TAMs in these processes allows for the development of therapeutic targets that inhibit metastasis through these mechanisms.

### 5.4. TIE2-Expressing Macrophages

A unique set of macrophages in the TME express the Tek tyrosine kinase receptor TIE-2 and have been deemed TIE-2 expressing macrophages (TEM). These macrophages commonly exhibit an M2 phenotype and are part of a distinct functional group that induces angiogenesis and tumor growth once recruited to the TME [115]. Historically, TIE-2 was regarded as an endothelial cell specific receptor, but since, TEMs, endothelial progenitor cells and pericyte precursors have all been discovered to have these angiogenic receptors. TEMs constitute a subpopulation of TAMs that can be distinguished by their surface marker profiles of Tie2^+^/CD11b^+^ and their preferences for highly vascularized, viable tumor areas. Human surface markers of TEMs include CD45^+^CD11b^+^CD16^+^CD14^low^L-Selectin^−^CCR2^−^ [116], while in mice, the surface markers are Cd45^+^Cd11b^+^Gr-1^low/−^ [117].

TEMs were first discovered in human tumors in 2007 by Venneri et al. in kidney, colon, pancreas, lung, and soft tissue carcinomas. Similar discoveries were made in human breast cancer later. TEMs are highly angiogenic [116] and lymphangiogenic [118] cells that display immune-suppressive activity by secreting IL-10 and VEGF in large amounts and dampen in vitro, tumor-specific T-cell proliferation by tumor dendritic cells [119].

The angiopoietin-Tie signaling pathway was identified as a vascular-specific receptor tyrosine kinase pathway essential for vessel development. The angiopoietin-Tie system appears to assist in the later stages of vascular development under normal physiological conditions such as in endothelial cell survival and vascular stability, maturation, and assembly [120]. Tie1 does not bind directly to angiopoietins and has weak kinase activity, while Tie2 binds directly to angiopoietins and has strong kinase activity, resulting in the downstream activation of the PI3K/AKT and ERK pathways. Historically, Ang2 has been viewed as an antagonist to Ang1, competitively binding to TIE-2 and inhibiting angiogenesis. Later studies have shown than Ang2 can phosphorylate TIE-2, but at a weaker rate than Ang1 [121].

In normal physiology and mature vessels, Ang1 promotes the strong activation of the Tie2/PI3K/AKT pathway, while Ang2 expression is suppressed through AKT-mediated inhibition of the FOXO1 transcription factor responsible for Ang2 expression. When AKT activity is low, FOXO1 is activated, and Ang2 expression increases. Here, Ang2-activates Tie2 phosphorylation and compensates for the absence of Ang1. Expression of Ang2 can be increased during inflammation by TNF-α expression. In situations where Ang2 is highly expressed, Ang2 binds to Tie2 and maintains Tie2 phosphorylation in an autocrine manner [121]. In mature vessels, Ang2 expression is low, but it is increased in inflammatory and angiogenic settings. Ang2 expression in cultured endothelial cells is increased by TNF, VEGF, and hypoxia; therefore, in highly angiogenic and hypoxic environments such as the TME, Ang2 expression by macrophages is constitutively activated by autocrine activation of the Tie-2 receptor on TEMs, leading to aberrant activation of angiogenic pathways PI3K/AKT [122].

### 5.5. Resistance to Therapy

Improving anti-cancer therapies is one of the most challenging problems in current medicine. Resistance to chemotherapy is typically correlated with increased levels of IL-6 and prostaglandin E2 (PGE2), which are both inflammatory mediators that lead to the differentiation of M2 macrophages [2]. Using the chemotherapeutics agents cisplatin and carboplatin on 10 different cervical and ovarian cancer cell lines, it was shown that this induced IL-10-secreting M2 macrophages that had elevated pSTAT3 and decreased pSTAT1 and pSTAT6 [2]. M2 macrophages have been found to be more resistant to radiation therapy than M1 macrophages, proving to be challenging for the treatment of cancer, as most TAMs are of the M2 phenotype [123]. TAMs exhibiting an M2-phenotype also contribute to resistance to anti-angiogenic therapy by increasing the population of M2-like macrophages in the TME after anti-angiogenic therapies [124].

Immune checkpoint blockade therapy resistance can be partially attributed to TAMs. Immune checkpoints consist of a family of proteins on T-cell surfaces that interact with specific ligands on APCs or cancer cells, inhibiting TCR-mediated activation [125]. Immune checkpoint therapies have reached the forefront of tumor immunotherapy in recent years, but clinical efficacy is only obtained in some patients and some cancer types. Due to TAM expression of ligand molecules for checkpoint receptors such as PD-L1/2, anti-checkpoint ligand monoclonal antibodies can be sequestered by the TAM secreted ligand, rendering it ineffective [125].

TAMs in tumors treated with chemotherapy can occasionally improve treatment efficacy but more commonly aid chemoresistance through three mechanisms: increased recruitment of MDSCs, suppression of adaptive, anti-tumor immune responses, and activation of anti-apoptotic programs in cancer cells. Chemotherapy-induced tissue damage promotes recruitment of MDSCs through secretion of IL-34 and CSF-1 from the cancer cells [125]. Breast cancer treated with doxorubicin showed promotion of CCL2 production by stromal cells, leading to recruitment of CCR2^+^ monocytes and contributing to tumor relapse [126]. In a transgenic mouse model of breast cancer, resistance to platinum-based therapy contributed to the downregulation of type I IFN-stimulated genes in TAMs [127]. Platinum therapy also induced the release of fatty acids on F4/80^+^/CD11low macrophages in the spleen, leading to the release of polyunsaturated lisophosphatidylcholines that altered the DNA damage response and led to resistance [127]. Paclitaxel or carboplatin treatment in mice with MMTV-PyMT tumors was counteracted by increased IL-10 secretion from TAMs, leading to downregulated IL-12 production in dendritic cells and the inhibition of CD8^+^ T-cell anti-tumor activity [128].

Due to the important role of TAMs in tumor angiogenesis, there is potential for interference with anti-angiogenic therapies. In tumors that were sensitive versus resistant to anti-VEGF therapy, a higher number of TAMs were found in patient samples of resistant tumors [129]. For example, anti-VEGF monoclonal antibody treatment can cause blood vessel damage, inducing hypoxia and stimulating the secretion of myeloid cell chemoattractants [128]. In combination therapy with anti-VEGF monoclonal antibodies and zoledronic acid, a therapy that deleted TAMs showed higher therapeutic efficacy than anti-VEGF therapy on its own in several different tumor models [130]. The Ang2–Tie2 pathway has also been targeted with a monoclonal antibody blocking the Ang2–Tie2 interaction in TEMs, reducing angiogenesis but increasing the recruitment of macrophages in murine breast and pancreatic cancer models [131].

Finally, radiation therapy has shown controversial results on TAMs. Glioblastoma tumors treated with X-ray radiation had an increased M2/M1 ratio of TAMs, but several other studies showed that low doses of radiation could reprogram macrophages towards an M1 phenotype [130].

Understanding the mechanisms of resistance of TAMs to therapy is fundamental to the development of therapeutic strategies to circumvent this resistive process and increase the efficacy of treatment without the risk of reoccurrence. Adjuvant anti-cancer therapies are being tested as targets of TAMs, and M2-targeted therapeutics have shown encouraging results in preventing metastasis and angiogenesis [88]. The complex heterogeneity of TAMs in the tumor microenvironment requires additional study. Understanding the role and mechanism of M2 TAMs in the TME is critical for understanding metastasis and the development of therapies to combat it and reprogramming the M2 macrophage toward an M1 phenotype is a promising therapeutic approach for cancer treatment [87].

### 5.6. Proposed Therapies

The complex biology of TAMs and their involved role in tumor proliferation, angiogenesis, EMT, metastasis, immune suppression, and therapy resistance makes developing anti-cancer therapies that consider all mechanisms of pro-tumor activity difficult. Some therapies aiming to either reprogram TAMs toward an M1 phenotype, kill existing TAMs, or inhibit the recruitment of new TAMs have been developed. CSF-1 has been described as the most important tumor-derived factor leading to monocyte recruitment through CCL2/CCR2 interaction, so CCR2 blockade therapies have been effective in suppressing TAM recruitment [132]. Both CCR2 inhibitors and anti-CCL2 monoclonal antibodies have been used in pre-clinical murine models to disrupt this CCL2/CCR2 interaction, showing efficacy both on their own and in combination with other anti-cancer therapies [133,134]. While this has shown promise, in murine breast cancer models, a rebound effect was shown after the withdrawal of anti-CCL2 treatment, increasing the infiltration of bone-marrow monocytes into the tumor and accelerating lung metastasis [135]. Another key pathway in monocyte recruitment and differentiation into TAMs is the CXCL12/CXCR4 interaction. In breast cancer, expression of CXCL12 by tumor cells increased the number of macrophages and blood vessel density, contributing to metastasis [136]. Inhibition of CXCR4 with AMD3100 reduced the formation of metastasis [137].

Depletion of TAMs is another therapy group being developed to help combat the multifaceted functions of TAMs in tumor progression and resistance. CSF-1 or CSF-1R expression in the TME has been associated with poor prognosis in many types of cancer. Because CSF-1 plays a key role in the proliferation and survival of monocytes and macrophages, the CSF-1/CSF-1R interaction is an attractive target for reducing the number of TAMs. A monoclonal antibody, emactuzumab, targets CSF-1R, decreasing the number of TAMs and increasing the CD8^+^/CD4^+^ T-cell ratio in the TME in a pre-clinical mouse model [138]. Small molecule CSF-1R blockades such as PLX3397 have been developed as well and have shown increased CD8^+^ T-cell infiltration and improved therapy response in murine models of several different tumor types [139]. While targeting the CSF-1/CSF-1R pathway appears to be an attractive target, some studies have shown that long-tern CSF-1R inhibition can lead to activation of the PI3K pathway and therapy resistance over time. A combination PI3K blockade and CSF-1R inhibition has shown positive results in pre-clinical trials [140].

Reprogramming M2 TAMs toward a more pro-inflammatory M1 phenotype is the third category of anti-TAM cancer therapy. Toll-like receptors are key players in M1 programming, as upon binding of a ligand to these receptors, macrophages are activated and exhibit an M1 phenotype [141]. Targeting of TLR3, TLR7, TLR8, and TLR9 have all been evaluated in the past few years but currently, a TLR7 agonist, imiquimod, is the only FDA-approved, topical-only treatment for squamous and basal cell carcinomas [142]. TLR3 stimulation with poly I:C has been shown to be more effective than imiquimod in reprogramming M2 TAMs to an M1 phenotype [143], but this type of treatment has not yet been fully developed. A nanoparticle containing poly I:C targeted for M2 TAMs was developed and in vitro, TNF-α and iNOS expression was upregulated and NO secretion was increased [144].

## 6. Conclusions

Tumor-associated macrophages play a key role in the development, metastasis, and reoccurrence of human malignancies, contributing to nearly every step of tumorigenesis. TAMs contribute to malignant cell proliferation, inflammation, host cell immunosuppression, angiogenesis and lymphangiogenesis, and therapy resistance. While there is still much to understand about macrophage regulation in the tumor microenvironment, it may prove to be a potentially effective anti-tumor therapeutic target if we gain the ability to control the switching of tumor-associated macrophages from M2 to M1 phenotypes.

## Figures and Tables

**Table 1:** Macrophage polarization markers on M0, M1 and M2 mouse and human macrophages.

Species	M0	M1	M2
**Mouse**	Csf1r, F4/80, CD11b	Marco, Cxcl9, Cxcl10, Cxcl11, Nos2, Socs1	Cd206, Tgm2, Fizz1, Chil3, Arg1, Ccl22, Cd163
**Human**	CSF1R, CD14, CD68, CD11B	CD86, MARCO, CXCL9, CXCL10, CXCL11, NOS2, SOCS1, CD64	TGM2, CD23, ARG1, CCL22, CD163, CD206

**Table 2:** Overview of extrinsic mechanisms of macrophage polarization.

Protein/Gene	Normal Function	Effect on Polarization
Interleukin-4 and Interleukin-13	Cytokines	M2-favored
Interleukin-4 receptor alpha	IL-4 and IL-13 signaling	M2-favored
Signal transducer and activator of transcription 6	Transcription factor	M2-favored
Peroxisome proliferator activated receptor gamma	Transcription factor	M2-favored
Tubular sclerosis 1	Inhibitor of mTOR	M2-favored
AKT Serine/Threonine Kinase 1	Signaling	M2-favored
AKT Serine/Threonine Kinase 2	Signaling	M1-favored
Src homology region 2 domain-containing phosphatase-1/2	Phosphatases	M1-favored
SH2-containing Inositol 5′-Phosphatase	Phosphatase	M1-favored
Phosphatase and tensin homolog	Lipid phosphatase	M1-favored
Myeloid differentiation primary response 88	Signaling adapter	M1-favored
Tumor necrosis factor	Cytokine	M1-favored
Tumor necrosis factor receptor 1	Cytokine receptor	M1-favored
Interferon-gamma, Interleukin-12	Cytokines	M1-favored

**Table 3:** Soluble factors secreted by M2-polarized TAMs, influencing various pro-tumor outcomes.

Factors Secreted by M2 TAMs	Pro-Tumorigenic Outcome
IL-6, EGF, TNF-α IL-8, IL-10, CCL2	Tumor growth
IL-10, TGF-β, MMP-7, PD-1, PDE-2, arginase	Immune suppression
CCL18, CCL22, MMPs, TGF-β, EGF, CCL20, IGF-1	Tumor invasion and metastasis
VEGFA, PDGF, COX2, HIF, MMPs, IL-10, adrenomedullin	Tumor angiogenesis and lymphangiogenesis
TGF-β, MMPs, IL-6, IL-10	Anti-cancer therapy resistance
